# Red and White Grape Pomace Possess Cardioprotective Effects by Modulating Inflammation and Oxidative Stress in Experimental Ischemic Heart Disease

**DOI:** 10.3390/molecules31020383

**Published:** 2026-01-21

**Authors:** Dan Claudiu Măgureanu, Raluca Maria Pop, Veronica Sanda Chedea, Paul-Mihai Boarescu, Mădălina Luciana Gherman, Ștefan Horia Roșian, Floricuța Ranga, Ioana Sorina Giurca, Elena Mihaela Jianu, Adriana Florinela Cătoi, Anca Dana Buzoianu, Ioana Corina Bocsan

**Affiliations:** 1 Pharmacology, Toxicology and Clinical Pharmacology, Department of Morphofunctional Sciences, “Iuliu Haţieganu” University of Medicine and Pharmacy, Victor Babeș, No 8, 400012 Cluj-Napoca, Romania; magureanu.dan@yahoo.com (D.C.M.); abuzoianu@umfcluj.ro (A.D.B.); bocsan.corina@umfcluj.ro (I.C.B.); 2 Research Station for Viticulture and Enology Blaj (SCDVV Blaj), 515400 Blaj, Romania; chedeaveronica@yahoo.com (V.S.C.); tirnovean.ioana@gmail.com (I.S.G.); 3 Department of Biomedical Sciences, Faculty of Medicine and Biological Sciences, “Stefan cel Mare” University of Suceava, 720229 Suceava, Romania; paul.boarescu@usm.ro; 4 Clinical Emergency County Hospital Saint John the New, 720229 Suceava, Romania; 5 Experimental Centre of “Iuliu Haţieganu”, University of Medicine and Pharmacy, Louis Pasteur, No 6, 400349 Cluj-Napoca, Romania; luciana.gherman@umfcluj.ro; 6 “Niculae Stăncioiu” Heart Institute Cluj-Napoca, 19-21 Calea Moților Street, 400001 Cluj-Napoca, Romania; dr.rosianu@gmail.com; 7 Department of Cardiology—Heart Institute, “Iuliu Haţieganu” University of Medicine and Pharmacy Cluj-Napoca, Calea Moților Street No. 19-21, 400001 Cluj-Napoca, Romania; 8 Food Science and Technology, Department of Food Science, University of Agricultural Science and Veterinary Medicine Cluj-Napoca, Calea Mănăștur, No 3-5, 400372 Cluj-Napoca, Romania; florica.ranga@usamv-cluj.ro; 9 Histology, Department of Morphofunctional Sciences, “Iuliu Haţieganu” University of Medicine and Pharmacy, Victor Babeș, No 8, 400012 Cluj-Napoca, Romania; jianu.mihaela21@gmail.com; 10 Pathophysiology, Department of Morphofunctional Sciences, Faculty of Medicine, University of Medicine and Pharmacy “Iuliu Hațieganu” Cluj-Napoca, 400012 Cluj-Napoca, Romania; adriana.catoi@umfcluj.ro

**Keywords:** white grape pomace, red grape pomace, polyphenols, inflammation, oxidative stress

## Abstract

**Background:** Cardiac ischemia (CI) remains a leading cause of death worldwide, prompting an ongoing search for new treatment options. This study explored and compared the preventive cardioprotective effects of polyphenols extracted from red (RGP) and white grape pomace (WGP) against isoproterenol (ISO)-induced myocardial ischemia, with a focus on their antioxidant and anti-inflammatory properties. **Materials and Methods:** Fifty male Wistar rats were divided into five groups: I—Saline, II—Saline+ISO, III—Ramipril+ISO, IV—WGP+ISO, and V—RGP+ISO. CI was induced in Groups II–V with ISO (45 mg/kg, on day 13), a dose widely used to reproducibly induce myocardial ischemic injury in experimental models. Electrocardiographic parameters, serum oxidative markers, cytokines, and tissue homogenates from the liver and heart were analyzed on day 14. **Results:** ISO significantly shortened the RR interval and increased the ventricular rate, without significant modulation by any treatment. The reduction in R-wave amplitude caused by ISO was lessened in all treated groups, with RGP showing values closer to Saline (RGP+ISO vs. Saline, *p* = 0.329). No differences were found among groups for PR segment, QRS duration, QT, or QTc intervals. Furthermore, all treated groups (III–V) showed significant improvements in oxidative and inflammatory markers compared to Saline+ISO (*p* < 0.05), with RGP demonstrating the strongest antioxidant activity by maintaining MDA and NO levels close to Saline (RGP+ISO vs. Saline, *p* > 0.05), while WGP exhibited superior anti-inflammatory effects in cardiac tissue by preserving IL-6 and IL-1β levels comparable to controls (WGP+ISO vs. Saline, *p* > 0.05). **Conclusions:** Grape pomace, especially RGP, may offer cardioprotection by decreasing oxidative stress, while WGP more effectively reduces inflammation. The complementary antioxidant and anti-inflammatory effects observed suggest that combining GP extracts may represent a promising hypothesis for future cardiovascular research.

## 1. Introduction

Cardiac ischemia (CI) is one of the most common pathological consequences of coronary artery disease and one of the most frequent clinical manifestations of cardiovascular disorders [1,2,3]. Coronary artery disease is characterized by periods of stability without evident manifestations or presenting as stable angina on exertion, and periods of instability, associated with a varied symptomatology, such as intense retrosternal pain, or even acute myocardial infarction [4]. CI is the leading cause of death worldwide, with the highest risk occurring in the population over 50 years old [2].

The primary etiology of this pathology is atherosclerosis; thus, a major treatment for the disease consists of changing the lifestyle to a healthy one with a decrease in lipid consumption and the abandonment of alcohol and smoking [5,6,7]. The pharmacological treatment of this disease is divided into two categories: secondary prevention for asymptomatic CI and medication to alleviate symptoms in symptomatic CI and to prevent complications [5]. Revascularization is performed through coronary bypass surgery or percutaneous coronary intervention in selected cases [8]. However, because these pharmacological and non-pharmacological interventions are associated with specific risks and adverse effects, there is a need to find an effective adjunctive therapy to reduce the risk factors of CI [5,9,10]. Despite advances in diagnostic strategies and pharmacological management, ischemic heart disease remains a leading cause of morbidity and mortality worldwide [11]. Therefore, taking into account the pathophysiological changes that characterize CI, namely oxidative stress and inflammation, new therapeutic options such as polyphenols from grape pomace (GP) could be used as adjunctive therapy to improve the mentioned changes [12,13,14]. These compounds are hypothesized to exert cardioprotective effects by modulating oxidative stress, inflammatory signaling pathways, endothelial function, and redox-sensitive cellular processes involved in myocardial injury. Although the antioxidant and anti-inflammatory effects of polyphenols from GP are well-established, there remains a lack of information regarding their pharmacological action in ischemic heart disease. In particular, comparative data addressing potential differences between red and white grape pomace in experimental models of cardiac ischemia are scarce. Therefore, further research is needed to clarify the mechanism of action and effects of polyphenols from GP.

While the cardioprotective, antioxidant, and anti-inflammatory properties of grape pomace and grape-derived polyphenols have been previously reported, the present study provides a novel comparative perspective by directly evaluating red versus white grape pomace within the same experimental model of isoproterenol-induced cardiac ischemia. In addition, this work differentiates their relative antioxidant and anti-inflammatory profiles and integrates biochemical findings with electrocardiographic indicators of myocardial injury, using ramipril as a pharmacological reference. This integrated approach allows a more nuanced interpretation of how compositional differences between grape pomace types may translate into distinct cardioprotective effects.

The objectives of this study are to evaluate the anti-inflammatory and antioxidant effect of polyphenols from white grape pomace (WGP) and red grape pomace (RGP) on isoproterenol (ISO)-induced CI, to evaluate the impact of GP on pro-inflammatory parameters—interleukin 1β (IL-1β), interleukin 6 (IL-6), and tumor necrosis factor alpha (TNF-α); oxidative stress markers—malondialdehyde (MDA), nitric oxide (NO), total oxidative stress (TOS), total antioxidant capacity (TAC), oxidative stress index (OSI), and total thiols (Thiols); and ECG parameters (RR segment, PR interval, QRS complex, QT and QTc intervals, ventricular rate, and amplitude of the R wave). In addition, the effects of grape pomace polyphenols were compared with those of the ACE inhibitor, ramipril.

## 2. Results

### 2.1. Grape Pomace Characterization

In the case of RGP extract, the total polyphenol content (TPC) was 25.90 ± 0.54 mg GAE/g, and the DPPH was 686.49 ± 17.83 mMTrolox/g d.w. GP (30 ± 0.05%I), while for WGP extract, the TPC was 38.80 ± 0.40 mg/g and the DPPH was 1069.02 ± 28.94 mMTrolox/g d.w. GP (54 ± 0.11%I). The results were in the range reported in the literature for both grape pomace extracts [12,13,15,16].

**Figure 1:**
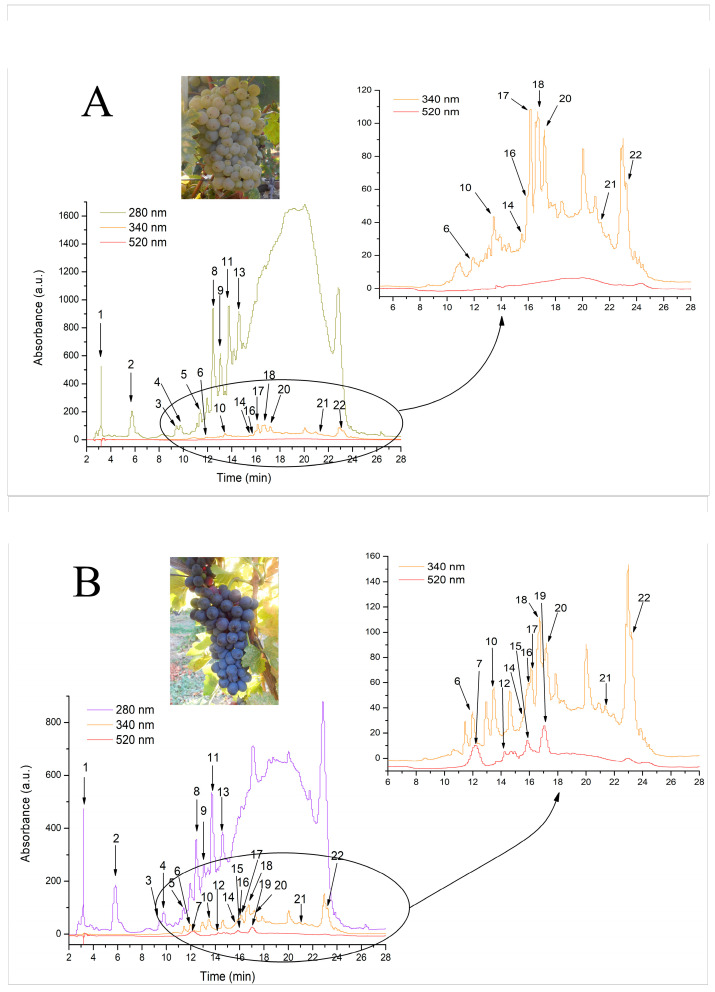
HPLC chromatogram of white grape pomace extracts obtained from white wine grapes (*Riesling Italian*) (**A**) and red wine grapes (*Amurg*) (**B**) as recorded at 280 nm, 340 nm, and 520 nm. Peak identification is presented in Table 1.

The phenolic compounds in GP samples were identified and quantified, with concentrations expressed as μg/g in the extract. A detailed presentation of the results can be found in Table 1 and Figure 1A,B. Flavanols represented the predominant phenolic subclass, accounting for approximately 87% and 75% of the total quantified phenolic content in WGP and RGP, respectively. Differences in the phenolic profile were further observed between the two GP varieties, with the presence of anthocyanins in the RGP (accounting for approximately 1.3% of total phenolics). Further, it was observed that total hydroxybenzoic acids and total hydroxycinnamic acids quantified in RGP were present at a ratio of approximately 2:1 compared with those in the WGP, as follows: 7.8% vs. 4.3% for hydroxybenzoic acids, and 6.5% vs. 3.6% for hydroxycinnamic acids, respectively. Flavonols were also present in both GP varieties, with 9% quantified in RGP and 5% in WGP.

### 2.2. Animal Groups Randomization

Rats were randomly assigned to the five experimental groups after baseline body weight measurement. Statistical analysis confirmed that there were no significant differences in baseline body weight among the groups, with data showing a normal distribution as assessed by Skewness (values between −1.00 and 1.00) and Shapiro–Wilk tests (*p* > 0.05) (Table A1). One-way ANOVA further confirmed the absence of statistically significant differences in mean body weight between groups at baseline (Table A2). To evaluate the potential effect of treatments on body weight, rats were reweighed on day 13; no significant differences were observed among groups, indicating that the treatments had no effect on body weight prior to ISO administration (*p* > 0.05) (Table A3).

### 2.3. Electrocardiography Findings

**Figure 2:**
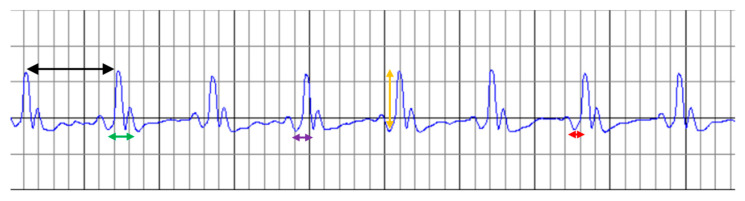
Measurements recorded on the ECG. Legend: black arrow—RR interval; green arrow—QT interval; purple arrow—QRS complex; orange arrow—R-wave amplitude; red arrow—PR segment. Physical parameters: The horizontal axis represents time (one small square = 40 ms), and the vertical axis represents voltage amplitude (one small square = 1 mm).

Figure 2 shows the ECG elements used to quantify treatment effects with ramipril, WGP, and RGP in ISO-induced myocardial infarction. Thus, the RR intervals, QT interval, PR segment, QRS complex, and R-wave amplitude were recorded on the ECG. In addition, based on these, the corrected QT interval and ventricular rate were calculated, and all of these were then compared between the five groups studied.

**Figure 3:**
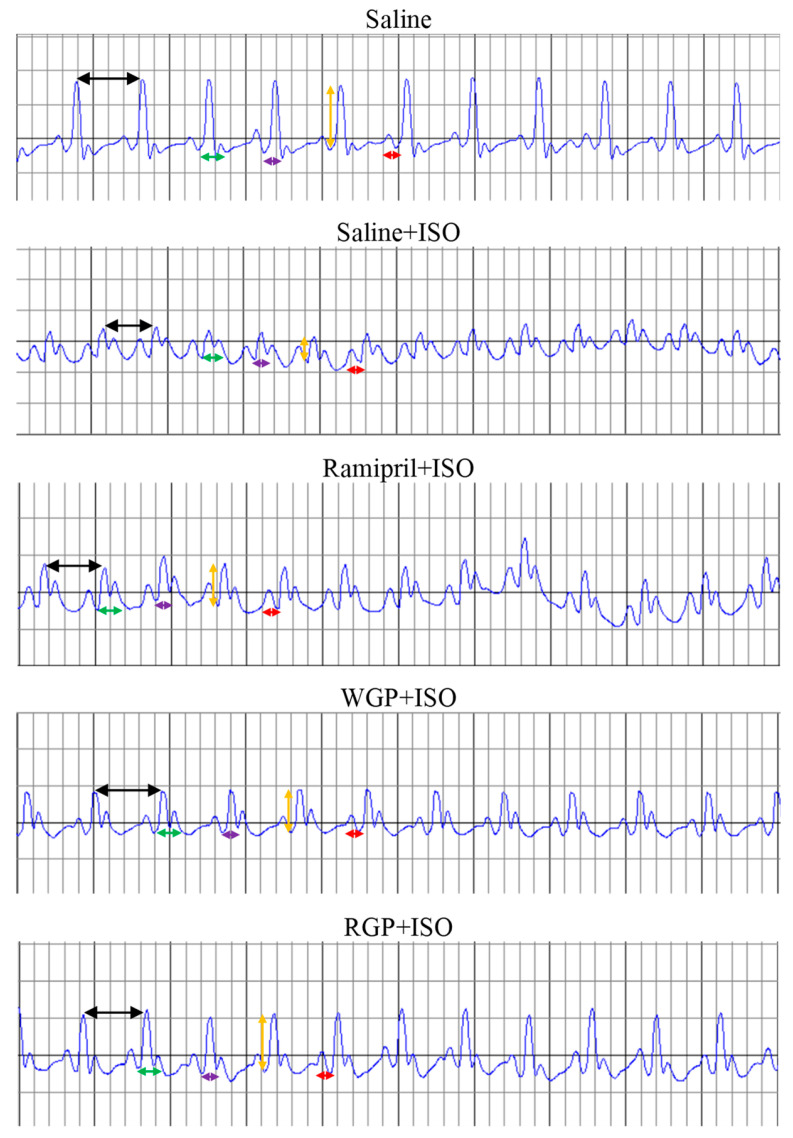
Representative ECG recordings from one animal in each experimental group. Legend: black arrow—RR interval; green arrow—QT interval; purple arrow—QRS complex; orange arrow—R-wave amplitude; red arrow—PR segment. Physical parameters: The horizontal axis represents time (one small square = 40 ms), and the vertical axis represents voltage amplitude (one small square = 1 mm).

Table 2 and Figure 3 show the ECG parameters characteristic of each group. Considering RR interval, as expected, ISO administration led to its decrease, while none of the three treatments prevented this effect (Saline+ISO vs. Ramipril+ISO, *p* = 0.067; Saline+ISO vs. WGP+ISO, *p* = 0.649; Saline+ISO vs. RGP+ISO, *p* = 0.292). In the case of ventricular rate (VR), the administration of ISO led to a marked increase in it, whereas none of the administered treatments exerted effects on the heart rate. Regarding the PR segment, the QRS complex, QT, and corrected QT intervals, no statistical differences were identified between the five tested groups (*p* > 0.05).

On the other hand, the most significant changes were observed in the amplitude of the R-waves. Thus, ISO led to a marked decrease in the amplitude of the R wave compared to that measured in the Saline group (*p* < 0.001). In the group preventively treated with ramipril, an improvement in the amplitude of the R waves was observed, but not statistically significant (Ramipril+ISO vs. Saline+ISO, *p* = 0.093), while between the Ramipril+ISO and Saline groups, the amplitude of the R waves was more significantly preserved statistically (*p* < 0.001). In the WGP-treated group, the reduction in R-wave amplitude was significantly attenuated compared with the Saline+ISO group (*p* = 0.002), indicating a superior protective effect to that of ramipril, especially since no significant difference was observed between the Ramipril+ISO and Saline+ISO groups (*p* = 0.141). However, WGP did not provide complete protection, as the R-wave amplitude still differed significantly from that of the Saline group (*p* = 0.021). The only preventive treatment that maintained the amplitude of the R waves similar to those in the Saline group was RGP, in which no statistical differences were identified (*p* = 0.329). This treatment showed statistically superior effects to ramipril (*p* = 0.010) but was not statistically significant compared to WGP (*p* = 0.232) (Table 2).

### 2.4. Antioxidant Serological Findings

Table 3 presents the effects of ISO and treatment administration on oxidative stress markers, as well as the antioxidant activity measured in serum samples from each experimental group. It was observed that ISO significantly increased MDA levels in all groups (Saline vs. Saline+ISO, *p* < 0.001; Saline vs. Ramipril+ISO, *p* = 0.002; Saline vs. WGP+ISO, *p* < 0.001), except the RGP group. Within this group, RGP administration-maintained MDA values comparable to those in the Saline group (*p* = 0.509). ISO also significantly increased NO levels (Saline vs. Saline+ISO, *p* < 0.001), with RGP again being the only treatment that maintained levels comparable to the Saline (*p* = 0.143). Furthermore, TOS levels increased in all groups, and the treatments did not maintain them at low levels. Next, ISO decreased TAC, but all treatments maintained values comparable to the Saline group (vs. Ramipril+ISO, *p* = 0.085; vs. WGP+ISO, *p* = 0.617; RGP+ISO, *p* = 0.239). As expected, ISO also increased OSI. While ramipril prevented this rise (Ramipril+ISO vs. Saline+ISO, *p* = 0.048), it did not reduce OSI to a level statistically comparable to that of the Saline group (Ramipril+ISO vs. Saline, *p* = 0.002). Finally, ISO did not significantly decrease total thiol levels (Saline vs. Saline+ISO, *p* = 0.083). However, both RGP and WGP significantly increased total thiol levels compared to the Saline+ISO group (vs. WGP+ISO, *p* = 0.004; RGP+ISO, *p* < 0.001). Total thiol levels were also higher than those of the Saline group, reaching statistical significance for RGP (*p* = 0.023) but not for WGP (*p* = 0.22).

### 2.5. Anti-Inflammatory Serological Findings

**Figure 4:**
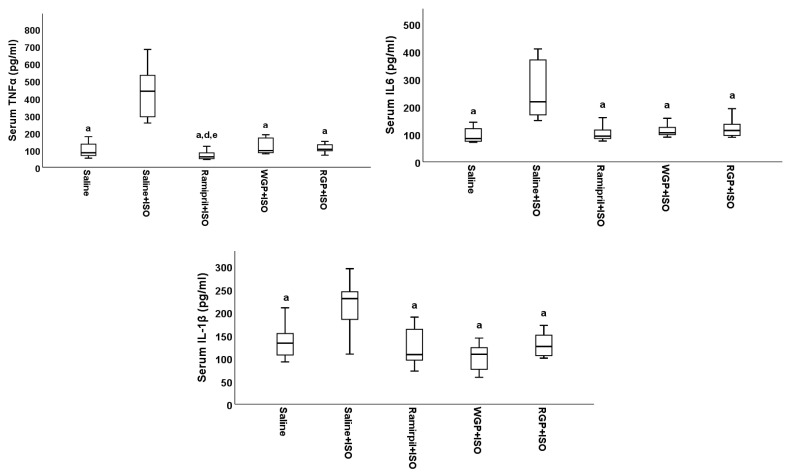
The effects of isoproterenol as quantified in serum by TNF-α, IL-6, and IL-1β. a had *p* < 0.05 versus Saline+ISO, d had *p* < 0.05 versus WGP+ISO, e had *p* < 0.05 versus RGP+ISO.

On day 14, after CI was induced, the serum TNF-α, IL-6, and IL-1β levels were quantified. As can be seen in Figure 4 and Table A4, 24 h after ISO administration, in the case of all three markers, statistically significant differences were identified between the values measured in the Saline, Ramipril+ISO, WGP+ISO, and RGP+ISO groups and those recorded in the Saline+ISO group (*p* < 0.05). Ramipril, WGP, and RGP had a marked anti-inflammatory effect, with no statistically significant differences in IL-6 and IL-1β values between the Ramipril+ISO, WGP+ISO, and RGP+ISO groups, respectively, and these and the Saline group (*p* > 0.05). Similar changes were also observed in the case of TNF-α values, except for a stronger anti-inflammatory effect induced by treatment with ramipril. In this case, TNF-α values were significantly lower compared to those measured in the WGP+ISO group (*p* = 0.014) and the RGP+ISO group (*p* = 0.020).

### 2.6. Anti-Inflammatory Findings in Heart and Liver Tissue Homogenates

**Figure 5:**
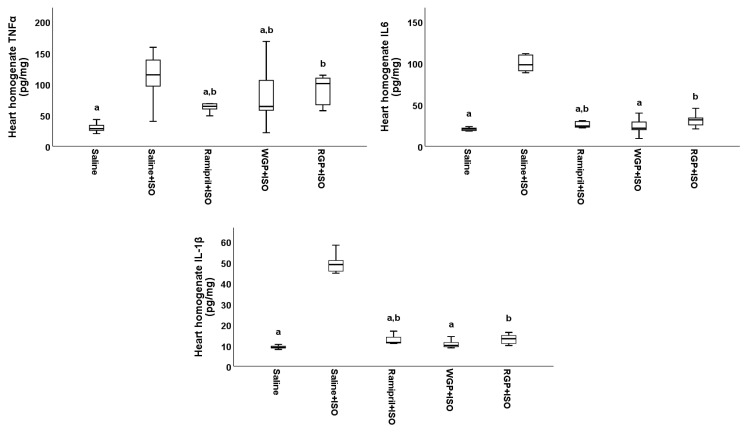
The effects of isoproterenol as quantified via heart homogenates, TNF-α, IL-6, and IL-1β. a had *p* < 0.05 versus Saline+ISO, b had *p* < 0.05 versus Saline.

**Figure 6:**
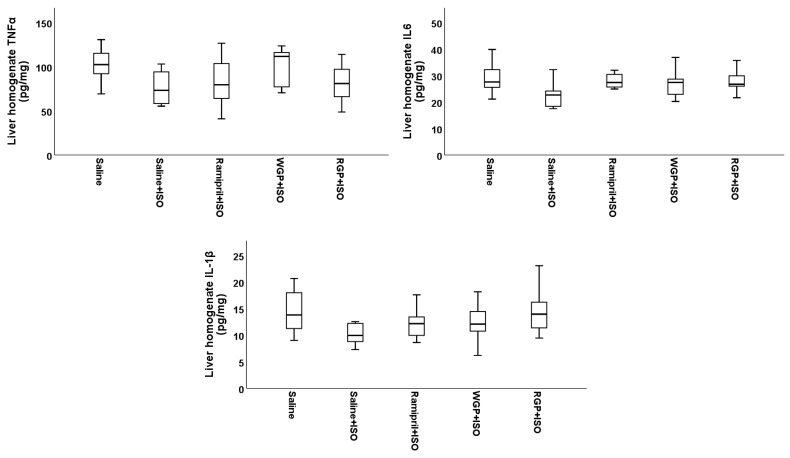
The effects of isoproterenol as quantified via liver homogenates, TNF-α, IL-6, and IL-1β.

Similar to the determinations in serum, to quantify the anti-inflammatory effects of WGP and RGP, the levels of TNF-α, IL-6, and IL-1β cytokines were measured in the heart (Figure 5, respectively, Table A5) and liver homogenates (Figure 6, respectively, Table A6). It was observed that the levels of TNF-α in the heart homogenate measured in the Ramipril+ISO and WGP+ISO groups were significantly lower as compared to the Saline+ISO group (*p* < 0.05) (Figure 5). Thus, preventive administration of ramipril and WGP prevented local TNF-α pro-inflammatory marker increase. Still, the anti-inflammatory effect was not potent enough since TNF-α values were significantly higher compared to the Saline group (*p* < 0.05). Administration of RGP could not prevent the increase in TNF-α levels in the heart tissue. Accordingly, no statistically significant differences (*p* = 0.44) between the RGP+ISO and Saline+ISO groups were observed. In the case of pro-inflammatory cytokines IL-6 and IL-1β, similar anti-inflammatory trends were evidenced. Accordingly, WGP administration prevented heart tissue IL-6 and IL-1β increase, and no significantly higher values were evidenced compared to the Saline group (for IL-6, *p* = 0.27, respectively, for IL-1β, *p* = 0.21) (Figure 5).

Pro-inflammatory cytokines TNF-α, IL-6, and IL-1β from hepatic homogenates had no statistically significant differences between the tested groups (*p* > 0.05) (Figure 6).

## 3. Discussion

The choice of the Italian Riesling and Amurg cultivars in the present study is motivated by the high cultivation share of *Italian Riesling* in the Târnave and Aiud vineyards, where it represents a substantial proportion of the planted vineyard area, as well as by the increasing agronomic importance of the autochthonous *Amurg* cultivar in Transylvania. In addition, the selected harvest period reflects recent climate-driven extensions of the active vegetation phase, which favor enhanced polyphenol accumulation at technological maturity and increase the oenological relevance and valorisation potential of the resulting winemaking by-products, particularly grape pomace [17,18,19]. Consequently, the phytochemical characterization of the resulting grape pomace is essential, as its biological effects are closely linked to compositional variability driven by grape variety, harvest conditions, vinification practices, and extraction procedures. In our study, WGP exhibited a moderately higher total phenolic content than RGP, whereas RGP was characterized by the presence of anthocyanins and relatively higher amounts of certain flavonols and phenolic acids, despite its lower overall TPC. Similar patterns have been reported in the literature, where red pomaces are typically richer in anthocyanins and flavonols, and white pomaces tend to have higher levels of flavanols and procyanidins, especially when seed content is greater [20,21]. For example, Radulescu et al. (2024) found that Romanian red pomaces were characterized by abundant anthocyanins and flavonols, whereas white pomaces exhibited higher catechin and epicatechin levels [20]. Likewise, Onache et al. (2022) showed notable differences in TPC and phenolic subclasses between white and red varieties, even when harvested under similar conditions [21]. These findings confirm that GP remains a concentrated source of bioactive phenolics, although the specific phenolic profiles vary widely among cultivars. Within the present study, the higher TPC observed in WGP compared with RGP can be explained by several methodological and biologically plausible explanations. Differences in grape cultivar, climatic and soil conditions, and pomace composition, such as skin-to-seed ratio, are well known to influence phenolic levels, as demonstrated in comparative analyses of red and white grape pomaces [20,21]. Fermentation-related factors, including maceration time and temperature, can modulate anthocyanin stability, leading to partial degradation during fermentation and resulting in lower residual anthocyanin content and total phenolics in the recovered pomace [22,23,24]. Similarly, drying temperature and extraction solvent significantly impact extraction efficiency and phenolic yield: higher drying temperatures and certain drying methods can markedly reduce anthocyanin and other phenolic contents, and solvent composition, like polarity and acidification, alters both total phenolic recovery and the relative yields of flavanols versus anthocyanins, depending on the extraction protocol used [25]. Seed-rich pomaces may exhibit higher TPC because grape seeds contain the highest concentrations of catechins and proanthocyanidins, which aligns with reports identifying seeds as the dominant phenolic source within GP [26,27]. These methodological differences may explain why WGP in this study presented higher overall TPC, whereas RGP demonstrated stronger activity on specific oxidative stress markers. Altogether, these data suggest that the qualitative phenolic composition, particularly the distribution of anthocyanins, flavanols, and proanthocyanidins, rather than total phenolic content alone, may contribute to a more favorable inflammatory and oxidative profile, thereby supporting protection against cardiovascular adverse events.

Importantly, the distinct phytochemical profiles identified in white and red grape pomace provide a mechanistic basis for the differential biological effects observed in this study. The higher flavanol and procyanidin content in WGP is consistent with its stronger anti-inflammatory activity, whereas the greater abundance of anthocyanins and specific flavonols in RGP aligns with its superior antioxidant effects and enhanced preservation of electrocardiographic parameters. Thus, the observed modulation of oxidative stress markers, inflammatory cytokines, and ECG alterations appears to be closely linked to the qualitative composition of grape pomace polyphenols rather than to total phenolic content alone.

ISO, a beta-adrenergic agonist, exhibits both positive inotropic and chronotropic activity. These effects, when administered at high doses, can lead to inflammation, myocardial hypertrophy, necrosis, and, ultimately, cardiac fibrosis [28,29]. Since prolonged or severe cardiac ischemia represents a pathological condition that can progress to the adverse event of acute myocardial infarction, ISO-induced injury is widely used to reproduce this transition in experimental models. Histopathological examinations conducted on rats after ISO administration revealed structural and functional changes at the cardiac level, similar to those found in acute myocardial infarction in humans [30].

The cardiotoxic effects of isoproterenol are primarily reflected by characteristic alterations on the ECG, which remains a key tool for assessing both injury and potential cardioprotective interventions. As ECG changes represent essential diagnostic elements of ischemia progressing to acute myocardial infarction, together with specific serological markers, they are widely used to document structural and functional myocardial impairment in ISO-induced models [31].

The literature has shown that isoproterenol induces multiple changes in the electrical conduction of cardiomyocytes, changes comparable to those evidenced in myocardial infarction. Thus, Moradi-Arzeloo et al. (2016) observed in an experimental model of myocardial infarction induced in rats that isoproterenol significantly increases the VR, associated with a significant decrease in R-wave amplitude, without observing changes in the QT interval [32]. A similar effect on R-wave amplitude was also observed in the study of Khorrami et al. (2014), in which infarction was induced using a dose of 100 mg/kg isoproterenol [33]. Using a similar experimental model, Periasamy et al. (2011) evidenced a marked increase in ventricular rate, QRS complex, and PR and QT intervals [34]. In the present study, the administration of a single dose of isoproterenol at 45 mg/kg induced a decrease in RR interval, an increase in ventricular rate, also attributed to the decrease in RR interval, and a decrease in R-wave amplitude, without identifying changes in PR segments, QT intervals, corrected QT, and QRS complexes. These changes can be explained based on the agonist effects on β-1 receptors exerted by isoproterenol. Thus, on the RR interval, none of the three preventive treatments had any protective effect, and this interval remained decreased in all four experimental groups. In the case of ventricular rate, the only treatment that showed cardioprotective activity was ramipril, while treatments based on white and red grape pomace determined modest changes. On the other hand, regarding the R-wave amplitude, a change that represents an electrocardiographic criterion for the diagnosis of acute myocardial infarction, the preventive treatment with ramipril did not present a satisfactory response to be considered as having a cardioprotective effect. Compared to this, the treatment with WGP showed a superior effect in maintaining the R-wave amplitude, but only RGP fully preserved the R-wave amplitude at values comparable to those of non-infarcted animals, thus suggesting that this treatment is the most effective in preventing a possible myocardial infarction. A recent study published in the literature by Pop et al. (2025) investigated, among other aspects, the cardioprotective effects of WGP extract, quantified through electrocardiography, on myocardial infarction induced by intraperitoneal administration of 45 mg/kg isoproterenol [35]. The study observed that while isoproterenol led to a prolongation of the QT and QTc intervals and a reduction in R-wave amplitude, the administration of WGP extract significantly prevented these changes. In our analysis, WGP contained substantially higher total polyphenol levels than RGP (38.80 vs. 25.90 mg/g) and demonstrated greater antioxidant activity (DPPH 54% vs. 30%). It also showed higher concentrations of catechin, epicatechin, and several procyanidin dimers, compounds recognized for strong antioxidant and anti-inflammatory effects (see Table 1). These flavanols are known to reduce ROS production, stabilize cardiomyocyte membranes, and preserve ion-channel function during stress, thereby limiting repolarization abnormalities and preventing the ECG changes typically induced by ISO [36,37]. This profile may partly explain the protective ECG effects observed with WGP. However, our results indicate that RGP elicited an even stronger response in maintaining the R-wave amplitude. This difference may relate to the distinct biochemical activity of the two extracts. RGP demonstrated a more pronounced ability to reduce oxidative stress, evidenced by the significant attenuation of ISO-induced elevations in MDA and NO, suggesting superior protection of cardiomyocyte membrane lipids and redox-sensitive ion channels. Given that oxidative injury and membrane destabilization directly contribute to reductions in depolarization amplitude, the capacity of RGP to limit lipid peroxidation and preserve membrane integrity offers a plausible mechanistic explanation for its superior preservation of the R-wave [38]. Together, these observations support the hypothesis that RGP’s stronger membrane-protective and antioxidant activity helps maintain normal depolarization amplitude even under ISO-induced stress.

Importantly, the electrocardiographic alterations observed in the present study can be interpreted in close relation to the biochemical changes associated with oxidative stress and inflammation. Oxidative damage and pro-inflammatory cytokine activation are known to impair cardiomyocyte membrane integrity, alter ion-channel function, and disrupt electrical conduction, thereby contributing to reductions in depolarization amplitude and other ECG abnormalities during ischemic injury. In this context, the concomitant attenuation of oxidative stress markers and inflammatory cytokines provides a coherent pathophysiological framework linking the observed preservation of R-wave amplitude with the improved redox and inflammatory status induced by grape pomace treatments.

Beyond their effects on cardiac electrical stability, as reflected by ECG parameters, these compositional differences between RGP and WGP also influence their antioxidant potential, an aspect highly relevant given the central role of oxidative stress in CI. Based on studies in the specialized literature, the prooxidant status induced by CI is very well-known based on multiple markers of oxidative stress, such as MDA levels [39,40,41], NO levels [42,43], TAC [44,45,46], TOS [47,48], OSI, and thiols levels [49,50,51]. The antioxidant properties of GP are well-documented and linked to its rich phenolic composition, with evidence of their effects also in CI pathologies [52,53,54,55]. Several studies have shown that grape-derived polyphenols reduce lipid peroxidation, modulate NO bioavailability, and enhance endogenous antioxidant enzymes in ISO-induced myocardial injury, contributing to significant reductions in MDA and NO [38,56]. Although no prior studies have directly compared RGP and WGP, the literature describing their typical phenolic profiles provides a mechanistic rationale that aligns with our findings. RGP, which generally contains higher concentrations of anthocyanins and certain flavonols, includes compounds with potent membrane-protective and lipid-peroxidation-inhibiting properties, which likely explains its superior ability to attenuate MDA elevation in our study [57,58]. Additionally, anthocyanins are known to influence both endothelial and inducible nitric oxide synthase activity, offering a plausible mechanism for the more effective normalization of NO levels observed with RGP [59]. In contrast, WGP, characterized by higher levels of flavanols such as catechin, epicatechin, and procyanidins, is often associated with improvements in global antioxidant buffering capacity, particularly through GSH-dependent pathways [60]. This profile corresponds well with our observation that TAC values did not differ significantly between RGP and WGP, suggesting that both extracts enhanced overall antioxidant capacity to a similar degree, despite exhibiting distinct effects on specific oxidative stress markers MDA and NO. To effectively utilize the distinct benefits of RGP and WGP, further investigations are required to understand the specific differences in their antioxidant profiles. Unfortunately, to our knowledge, no studies have compared the antioxidant effects induced by RGP versus WGP. However, this study reported similar antioxidant effects in terms of TAC, while RGP showed a clearly stronger activity in reducing ISO-induced elevations in MDA and NO, maintaining both markers at levels close to the control group. Additionally, both RGP and WGP significantly increased total thiol levels compared to the control group, a finding consistent with prior reports demonstrating that grape pomace polyphenols strengthen thiol-based antioxidant systems and reduce oxidative protein modification [61,62]. It should be noted that thiol status was assessed as total thiol content; therefore, potential shifts between reduced thiols and disulfide forms could not be evaluated and would require targeted analyses of thiol–disulfide homeostasis in future studies.

Positive correlations have been established between acute myocardial infarction and elevated levels of pro-inflammatory cytokines such as TNF-α, IL-6, and IL-1β [63,64,65,66]. Although these cytokines can, to a certain extent, contribute to the initiation of cardiac repair, their excessive and prolonged activation can trigger an exaggerated inflammatory response associated with delayed and maladaptive tissue repair, ultimately resulting in excessive fibrous tissue formation and impaired cardiac function [28,63]. Thus, the levels of these cytokines can be analyzed to determine the intensity and duration of the inflammatory process in the myocardial infarction. Furthermore, several studies have shown that a marked increase in the levels of these cytokines above the already elevated levels in patients who have recently suffered a myocardial infarction can be a predictive factor for the development of acute heart failure [64,65]. Additionally, because IL-6 is generated at the endothelial level in cases of inflammation or oxidative stress, and given the observation of a statistically significantly higher level of this marker at the coronary level compared to the arterial level, a higher level of IL-6 has been associated with a negative prognosis in various pathologies [66].

Thus, the increase in these pro-inflammatory cytokines, TNF-α, IL-6, and IL-1β, induced by ISO has been demonstrated in multiple specialized studies in the literature [67,68,69,70,71,72,73]. In this study, results aligned with the existing literature, showing a similar inducing effect on cytokine expression. Among the observed changes, TNF-α exhibited the most significant increase, with its average level rising more than fourfold following ISO administration compared to pre-treatment levels. However, preventive treatments with ramipril, as well as WGP and RGP, successfully maintained TNF-α levels comparable to those in non-infarcted rats. This suggests a potential anti-inflammatory and cardioprotective effect in the context of CI. Similarly, ISO administration led to a twofold increase in IL-6 and IL-1β levels. Yet, all three treatments, ramipril, WGP, and RGP, exerted comparable anti-inflammatory effects, demonstrating similar efficacy in reducing these markers. A similar effect was observed in the study of Giribabu et al. (2016), who investigated the effect of the methanolic extract of *Vitis vinifera* seeds on an experimental model of myocardial infarction induced by ISO in Wistar rats [74]. They demonstrated the same anti-inflammatory and cardioprotective effect, as demonstrated by the inhibition of the increase in the three markers studied. However, we did not identify any more studies on this topic in the specialized literature, nor studies that directly compare the effects induced by WGP and RGP.

The pro-inflammatory effects of ISO have also been directly demonstrated at the level of cardiac tissue, with significant increases in these cytokines being observed [75,76,77,78,79,80,81]. In the present study, data comparable to those in the literature were observed, with ISO inducing a nearly four-fold increase in TNF-α, IL-6, and IL-1β measured in cardiac homogenates. Among the tested interventions, ramipril and WGP partially attenuated this local inflammatory response, while WGP was the only preventive treatment that maintained IL-6 and IL-1β levels comparable to those of the control group, highlighting a tissue-specific anti-inflammatory effect. In contrast, TNF-α levels in cardiac tissue remained significantly elevated compared to the control group. Regarding hepatic homogenates, we did not find studies that evaluated the effect of isoproterenol on TNF-α, IL-6, or IL-1β levels. In our experiment, cytokine values did not differ between animals receiving ISO alone and those receiving ISO together with the tested treatments. These findings suggest that, within 24 h after ISO administration, the liver may not exhibit an acute inflammatory response to this dose. However, this could be explained by the fact that in this study, a single dose of 45 mg/kg body weight of ISO was administered, a dose lower than that required to manifest effects on hepatic tissue. Comparatively, in the literature, a hepatotoxic effect of isoproterenol has been reported at a dose of 85 mg/kg body weight administered over two consecutive days [82].

Although the present findings are consistent with several experimental studies reporting antioxidant and anti-inflammatory effects of grape-derived polyphenols, it should be acknowledged that not all investigations have demonstrated uniform cardioprotective outcomes. Variability in grape cultivar, extraction methods, dosing regimens, and experimental models may contribute to divergent results across studies. Moreover, while experimental models such as isoproterenol-induced cardiac injury provide valuable mechanistic insights, their translational relevance to human ischemic heart disease remains inherently limited. These considerations underscore the need for cautious interpretation of the present findings and highlight the importance of further studies addressing dose–response relationships, long-term effects, and clinical applicability. Finally, while the antioxidant and anti-inflammatory potential of grape pomace polyphenols is well recognized, comparative data regarding total polyphenol content and biological activity depending on the source of pomace, white versus red grape pomace, remain limited. Therefore, future studies should systematically evaluate the influence of geographic origin, climatic conditions, and preparation methods using standardized analytical approaches to better define compositional and functional differences. Such efforts are necessary to support the rational valorization of grape pomace polyphenols for cardiovascular prevention. In addition, well-designed clinical studies comparing the effects of white and red grape pomace in humans are still needed to clarify their potential role in the prevention and management of ischemic heart disease.

## 4. Materials and Methods

### 4.1. Chemicals and Reagents

The isoproterenol (≥98.5% purity, catalog number I5627-5G, lot BCBW7995) used to induce acute myocardial infarction was purchased from Sigma-Aldrich (St. Louis, MO, USA). Ethanol (S120/022025, Chimreactiv SRL, Bucuresti, Romania), sodium carbonate (catalog number 497-19-8), acetic acid (≥99% purity, catalog number 83876.330), Folin–Ciocalteu reagent (catalog number 31360.264), and phosphate buffer were purchased from VWR Chemicals (Radnor, PA, USA) and Sigma Co. (St. Louis, MO, USA). HPLC-grade acetonitrile (catalog number 83639.320, lot 17F081391) from VWR Chemicals (Radnor, PA, USA) and ultrapure water (catalog number L0020, lot 0905A) were obtained from Biochrom (Cambridge, UK). Gallic acid (≥95% purity, catalog number 91215-100MG, lot BCCC0553), catechin (≥99% purity, catalog number 43412-10MG, lot BCCD1116), and rutin (≥94% purity, catalog number R5143-50G, lot BCBD8327V) were sourced from Sigma Co. (St. Louis, MO, USA).

### 4.2. Plant Material

Both red and white grape pomaces consisted of skins, seeds, and stems of red wine grapes (*Amurg*), respectively, of white wine grapes (*Riesling Italian*), provided by SCDVV Blaj winery (Blaj, TârnaveWine Center, România). The grape cultivars were harvested from Crăciunelul de Jos vineyard in October 2021. The white and red GP were obtained by pressing the grapes, which were dried at room temperature (23 °C, 50% relative humidity) in a well-ventilated room until constant weight was reached, confirming completion of the drying process. After drying, both RGP and WGP were frozen at −80 °C until the extraction.

### 4.3. Grape Pomace Extraction

An equal amount (250 g) of each RGP and WGP cultivar was finely ground and extracted using a 20/80 (*v*/*v*) H_2_O–ethanol mixture (3 L). The extraction process involved 30 min of sonication using a Witeg WiseClean WUC-D06H (40 kHz, 200 W, and at room temperature) from Witeg Labortechnik GmbH (Wertheim, Germany), followed by filtration using Whatman no. 3 filter paper. The pellets were re-extracted, and the supernatants were pooled, repeating the procedure three times. The resulting grape pomace extracts were then concentrated to 0.8 L using a rotary evaporator (Heidolph Hei-VAP Platinum 3, Heidolph Scientific Products GmbH, Schwabach, Germany) and subsequently analyzed for total polyphenol content and phenolic composition. The use of a 20/80 (*v*/*v*) H_2_O–ethanol solvent mixture was selected to align with green, food-grade processing, as ethanol and water are safe, environmentally friendly solvents widely accepted for food and nutraceutical applications. Also, water and ethanol allow the simultaneous extraction of both polar and moderately polar polyphenols. To avoid batch-to-batch variability, all extracts were prepared under the same conditions (identical solvent ratio, extraction time, and temperature). Also, the results were normalized to dry weight, while the composition was monitored through repeated HPLC profiling of major phenolic compounds to ensure extract standardization and comparability across batches.

### 4.4. Total Polyphenol Content (TPC)

The total phenolic content (TPC) was measured using the Folin–Ciocalteu method [83,84]. Briefly, 25 mL of WGP and RGP extracts were mixed with 125 mL of Folin–Ciocalteu reagent (0.2 N) and 100 mL of sodium carbonate (7.5% *w*/*v*). The mixtures were homogenized and incubated in the dark at room temperature for 2 h. Absorbance was recorded at 760 nm using a Synergy HT Multi-Detection Microplate Reader (BioTek, Winooski, VT, USA). TPC was quantified based on a gallic acid calibration curve (R^2^ = 0.9945) and expressed as gallic acid equivalents (GAE). Each sample was analyzed in triplicate, with results reported as mean values (mg/100 g DW) ± standard deviations. Although the Folin–Ciocalteu assay is widely used for estimating total phenolic content, it lacks specificity because it also responds to other reducing substances present in grape pomace extracts; therefore, this limitation was further addressed by complementing the analysis with HPLC-based profiling to achieve a more accurate and specific characterization of individual phenolic compounds.

### 4.5. Determination of DPPH Radical Scavenging Capacity

The radical-scavenging capacity of each GP extract was evaluated using the DPPH test. The extract was prepared from 250 g GP to a final volume of 0.8 L, corresponding to 0.3125 g dry weight GP/mL, and diluted 1:1000 prior to analysis. A mixture of 1750 μL DPPH solution (0.02 mg/mL in methanol) and 250 μL of each GP extract, yielding a final reaction volume of 2.0 mL and a final concentration of 0.039 mg GP/mL. The mixture was incubated at room temperature for 30 min. Absorbance at 517 nm was measured using a Synergy HT Multi-Detection Microplate Reader (BioTek Instruments, Inc., Winooski, VT, USA). Antioxidant activity was quantified using a Trolox calibration curve (r^2^ = 0.9985) and expressed as Trolox equivalent antioxidant capacity (µM Trolox/g GP). In parallel, the DPPH˙ radical scavenging activity of GP extracts was determined as the percentage of radical inhibition at the tested concentration using the following equation and expressed as mean values ± standard deviations (n = 3):

$$DPPH\;scavenging\;\left(\%\right)=\;\frac{Ac-As}{Ac}\times 100$$

*Ac*—control absorbance.
*As*—sample absorbance.

### 4.6. Analysis of Phenolic Compounds Using Liquid Chromatography-Diode Array Detection-Electro-Spray Ionization Mass Spectrometry (HPLC-DAD-ESI MS)

High-performance liquid chromatography (HPLC) is a widely used method for separating, quantifying, and identifying compounds in mixtures [85]. The HPLC-MS analysis of WGP ethanolic extract followed the method of Pop et al. (2022), using an Agilent 1200 HPLC system with DAD detection, coupled to an Agilent 6110 mass spectrometer (Agilent Technologies, Santa Clara, CA, USA) [86]. Separation occurred at room temperature on an Eclipse XDB C18 column (4.6 × 150 mm, 5 μm), with a mobile phase gradient of (A) 0.1% acetic acid/acetonitrile (99:1) in water and (B) 0.1% acetic acid in acetonitrile. The injection volume was 20 mL. The gradient started at 95% A, gradually decreasing to 10% A before returning to initial conditions. The flow rate was 0.5 mL/min, and absorbance was recorded at 280 and 340 nm. Compounds were detected via electrospray ionization (ESI) in positive ion mode, with a source temperature of 350 °C, nitrogen flow at 8 L/min, and a capillary voltage of 3000 V. Mass spectra were scanned over an *m*/*z* range of 100–1000, and data were processed using Agilent ChemStation Software (Rev B.04.02 SP1, Palo Alto, CA, USA). Compound identification was based on UV-visible spectra, retention times, and mass spectra. Following this, compounds belonging to hydroxybenzoic acids were quantified as gallic acid equivalent (R2 = 0.9978; y = 33.624x + 30.8; LOD = 0.35 ug/mL, LOQ = 1.05 ug/mL); compounds belonging to flavanols were quantified as catechin equivalent (R2 = 0.9985, y = 15.224x − 130.24, LOD = 0.18 μg/mL, LOQ = 0.55 μg/mL); and compounds from flavonols class as rutin equivalent (R2 = 0.9981, y = 26.935x − 33.784, LOD = 0.21 μg/mL, LOQ = 0.64 μg/mL). LOD and LOQ were calculated in Excel using the calibration-curve method (LOD = 3.3σ/S; LOQ = 10σ/S; S = slope; σ = residual SD of the regression).

### 4.7. Animals

The experiment included 50 male Wistar albino rats obtained from the biobase of the “Iuliu Hațieganu” University of Medicine and Pharmacy, Cluj-Napoca. The body weight of the animals ranged between 150 and 270 g at the beginning of the study. The animals were kept in optimal conditions: at a constant temperature (22 ± 2 °C), stable air humidity (45 ± 10%), and respecting the 12/12 h day–night cycle. Each group was placed separately in a polycarbonate cage, equipped with a grid on the top. The animals had standard food and water ad libitum.

Throughout the study, appropriate hygiene standards were observed, and at the end of the experiment, specific decontamination of the used space was carried out. Before the start of the study, the animals were randomized and divided into 5 equal groups of 10 animals each, homogeneous based on the previously determined weight. Twenty-four hours before the experiment, the animals were fasted, with water administered ad libitum.

### 4.8. Experimental Design

Each animal received the pharmacological substance for 13 days. CI was induced using isoproterenol administered on day 13. In accordance with ARRIVE guidelines, a Saline-treated control group was included to account for vehicle administration and procedural effects. Animals were assigned to control or treatment groups using a computer-generated randomization procedure, randomized according to body weight. Grape pomace extracts, Saline, and ramipril administration were conducted once daily in the morning at a fixed time. Isoproterenol-induced myocardial infarction was induced on day 13, two hours after Saline, extracts, and drug administration. Treatment administration and outcome assessments were performed by separate investigators blinded to group allocation. Finally, grape pomace, Saline, and ramipril were administered by gavage, while isoproterenol was administered by intraperitoneal injection, as follows:

- Group I (Saline) consisted of 10 rats weighing between 165 and 240 g. This was considered the control group, and the animals were administered 0.5 mL/100 g of Saline.
- Group II (Saline+ISO) consisted of 10 rats weighing between 160 and 260 g. Medication administered: 0.5 mL/100 g Saline and ISO 45 mg/kg body weight.
- Group III (Ramipril+ISO) consisted of 10 rats weighing between 160 and 260 g. Medication administered: ramipril 10 mg/kg body weight and ISO 45 mg/kg body weight.
- Group IV (WGP+ISO) consisted of 10 rats weighing between 160 and 250 g. Medication administered: white grape pomace and ISO 45 mg/kg body weight.
- Group V (RGP+ISO) consisted of 10 rats weighing between 150 and 270 g. Medication administered: red grape pomace and ISO 45 mg/kg body weight.

The doses of GP extracts were selected based on data from the literature from experimental myocardial infarction models [58,87,88]. Accordingly, the administered doses corresponded to 16.19 and 24.25 mg GAE/kg b.w. in rats receiving the RGP and WGP extracts, respectively. These doses fall within the low-to-moderate range of phenolic exposures reported to exert cardioprotective effects in preclinical models and were selected to avoid high doses of polyphenol administration.

### 4.9. Electrocardiography

CI was determined using ECG monitoring on day 14 of the experiment, the second day following the intraperitoneal injection of isoproterenol. During ECG recording, each rat was anesthetized by intraperitoneal injection of a ketamine–xylazine mixture to eliminate any artifact.

After inducing anesthesia, three electrodes were placed on each rat: one on each forelimb and one on the right hindlimb. The ECG was recorded using a Biopac MP36 system (BIOPAC Systems, Inc., Goleta, CA, USA), using the following calibration: 1 mV/1 cm and a speed of 50 mm/s. Using Biopac Student Lab 3.7.7 software, RR and QT intervals (ms), PR segment (ms), QRS complex duration (ms), and R-wave amplitude (mm) were recorded. The ventricular rate was calculated using the formula VR = 60,000/RR, and the corrected QT interval (QTc) was calculated using Bazett’s formula: QTc = QT/√RR [89].

### 4.10. Inflammatory Markers Analysis

Blood was collected from each animal to determine TNF-α, IL-1β, and IL-6 levels on day 14 of the experiment. Samples were collected via a puncture of the orbital sinus using a capillary tube or a sterile Pasteur pipette (to avoid subsequent superinfection). To limit the animal’s discomfort, a local anesthetic was administered a few minutes before the procedure. During the collection of biological samples, the animals were restrained by a manual method performed by personnel specifically trained in animal handling. An amount of 0.5–1 mL of blood was collected. This amount is acceptable since 10% of the total blood volume (55–70 mL/kg) can be collected weekly. The serum obtained by centrifugation of the samples was stored at −80 °C until the determinations were performed. Levels of the cytokines TNF-α (catalog no. 900-K73), IL-6 (catalog no. 900-K86), and IL-1β (catalog no. 900-K91) were measured using the ELISA technique (BioTek 50 TS Microplate Washer and BioTek 800 TS Microplate Reader – BioTek, Winooski, VT, USA), following the protocols provided by commercially available ABTS ELISA development kits (PeproTech EC, Ltd., London, UK). For each test, protein levels were calculated using a standard curve (4-PL).

### 4.11. Oxidative Stress Analysis

Oxidative stress status was evaluated by measuring total antioxidant capacity (TAC), total oxidative stress (TOS), malondialdehyde (MDA), nitric oxide (NO), and total thiols (THIOL) levels in blood samples. NO levels were measured using the Griess reaction after nitrate reduction by vanadium (III), as described by Miranda et al. [90]. MDA, a lipid peroxidation marker, was detected using the thiobarbituric acid method. Absorbance values at 532 nm were obtained after 30 min incubation and correlated with MDA concentrations [91]. Redox status was appraised using the oxidative stress index (OSI), calculated as OSI = TOS/TAC [92]. THIOL levels were determined using Ellman’s reagent [93]. Absorbance measurements were performed using a Jasco V-350 UV-VIS spectrophotometer (Jasco International Co. Ltd., Tokyo, Japan).

### 4.12. Tissue Homogenates

At the end of the experiment, the animals were euthanized by anesthetic overdose with a mixture of 2% xylazine and 10% ketamine. Subsequently, the liver and heart were surgically removed from each animal. The tissues were weighed and homogenized (at 27,000 rpm using a Witeg Homogenizer automatic HG-15D, Wertheim, Germany) in four volumes of phosphate-buffered Saline, then centrifuged at 15,000 rpm for 15 min at 4 °C. The resulting supernatant was used for further analysis. The protein content of the heart and liver samples was measured using the BCA kit (Pierce, Rockford, IL, USA) according to the manufacturer’s instructions. Levels of pro-inflammatory cytokines (TNF-α, IL-6, and IL-1β) were calculated relative to the total protein content of the sample (pg/mg).

### 4.13. Statistical Analysis

The results were statistically analyzed using SPSS v20. The data obtained in the experimental study were analyzed at each measurement time point and compared by comparing the mentioned time points, which were considered quantitative variables. Comparisons were made between the control and experimental groups and within the same group at the mentioned measurement time points. Skewness and Shapiro–Wilk tests were used to verify the normal distribution of the variables. In the case of normally distributed data, the mean and standard deviation were used for group comparison by the ANOVA test, while for data that did not present a normal distribution, the median and percentiles (25th–75th) were used by the Kruskal–Wallis test. The level indicating statistical significance was set at *p* < 0.05.

### 4.14. Research Ethics

This study adhered to the guidelines outlined in the “Guide for the Care and Use of Laboratory Animals” (National Institutes of Health, 2011). The protocol was approved by the Ethics Committee of the “Iuliu Haţieganu” University of Medicine and Pharmacy, Cluj-Napoca, and the Cluj-Napoca Veterinary Health Directorate (no. 298/01 April 2022).

## 5. Limitations of the Study

Firstly, an echocardiographic evaluation of left ventricular structure and function was not performed due to technical and logistical constraints related to the experimental setup and study design, although such an assessment would have provided additional insights into the effects of the treatments on cardiac performance. Moreover, histopathological analysis of ischemic myocardial areas was not conducted, limiting the direct assessment of myocardial injury extent and the comparison of lesion size and distribution among treatment groups. Histopathological evaluation could have complemented the biochemical and functional findings by allowing direct correlation between inflammatory and oxidative stress markers and structural myocardial damage. In addition, although ECG recordings allowed the evaluation of electrical alterations induced by ISO, advanced imaging techniques such as cardiac magnetic resonance imaging or other radiological modalities could offer a more comprehensive assessment of regional and global myocardial dysfunction. In the present study, ECG parameters together with circulating and tissue biomarkers of inflammation and oxidative stress represented the primary indirect measures used to assess myocardial injury, while additional compensatory approaches were not feasible within the scope of the experimental protocol. Finally, since isoproterenol was administered systemically, the resulting cardiac injury likely involved diffuse myocardial dysfunction rather than focal ischemic lesions, an aspect that should be considered when interpreting the findings. Also, another limitation of this study is the use of a single dose of grape pomace extract, which limited the evaluation of a dose–response relationship and the absence of recovery (spiking) experiments. Nevertheless, the present study represents an important step toward exploring the cardioprotective potential of red and white grape pomace and supports further investigations using complementary imaging-based approaches.

## 6. Conclusions

In conclusion, the present study demonstrates that grape pomace polyphenols, when administered preventively, exert measurable anti-inflammatory and antioxidant effects in an experimental model of isoproterenol-induced cardiac ischemia. These effects are strongly supported by the observed modulation of pro-inflammatory cytokines (TNF-α, IL-6, and IL-1β) and oxidative stress markers in serum and cardiac tissue. Notably, white grape pomace exhibited a slightly more pronounced anti-inflammatory effect in cardiac tissue, whereas red grape pomace showed superior antioxidant activity and a stronger ability to preserve R-wave amplitude, potentially reflecting differences in phenolic composition and redox-modulating properties.

While these findings support the cardioprotective potential of grape pomace at the experimental level, extrapolation to clinical settings should be made with caution, given the limitations of the present study. The observed complementary effects of red and white grape pomace suggest that combined approaches may warrant further investigation; however, additional preclinical studies incorporating advanced imaging and histopathological analyses, followed by well-designed clinical trials, are required before any clinical application or therapeutic recommendation can be considered.

## Figures and Tables

**Table 1:** Identification and quantification of the phenolic compounds of the red (RGP) and white grape pomace (WGP).

Peak No.	R_t_ (min)	UV λ_max_ (nm)	[M + H]^+^ (*m*/*z*)	Phenolic Compound	Subclass	WGP Concentration (μg/g)	RGP Concentration (μg/g)
1	3.20	270	139	2-Hydroxybenzoic acid	Hydroxybenzoic acid	114.98 ± 5.75	127.47 ± 6.38
2	5.76	270	171	Gallic acid	Hydroxybenzoic acid	220.52 ± 11.03	198.92 ± 9.95
3	9.47	280	579	Procyanidin dimmer B3	Flavanol	206.97 ± 7.07	91.67 ± 5.37
4	9.79	290, 260	155	Protocatechuic acid	Hydroxybenzoic acid	83.77 ± 3.77	61.83 ± 3.71
5	11.41	280	579	Procyanidin dimmer B1	Flavanol	320.09 ± 17.60	168.84 ± 6.75
6	11.97	332	343	Caffeic acid-glucoside	Hydroxycinnamic acid	381.89 ± 22.91	372.76 ± 16.77
7	12.18	530, 280	493	Malvidin-glucoside	Anthocyanin	n.d.	23.15 ± 1.16
8	12.45	280	291	Catechin	Flavanol	2246.87 ± 89.88	854.46 ± 51.27
9	13.07	280	579	Procyanidin dimmer B4	Flavanol	1430.04 ± 71.50	603.48 ± 24.14
10	13.45	360, 250	435, 303	Quercetin-arabinoside	Flavonol	46.38 ± 2.78	85.71 ± 4.71
11	13.72	280	291	Epicatechin	Flavanol	2218.59 ± 106.49	1319.78 ± 68.63
12	14.26	530, 280	535	Malvidin-acetyl-glucoside	Anthocyanin	n.d.	9.13 ± 0.55
13	14.61	280	579	Procyanidin dimmer B2	Flavanol	2559.34 ± 115.17	1258.64 ± 62.93
14	15.52	360, 250	611, 303	Quercetin-rutinoside (Rutin)	Flavonol	42.99 ± 1.72	13.89 ± 0.76
15	15.85	530, 280	655	Malvidin-diglucoside	Anthocyanin	n.d.	19.049 ± 0.95
16	15.95	360, 260	303	Ellagic acid	Hydroxybenzoic acid	35.80 ± 2.15	55.74 ± 2.51
17	16.15	360, 250	465, 303	Quercetin-glucoside	Flavonol	100.44 ± 5.22	78.87 ± 5.78
18	16.71	360, 260	479, 317	Isorhamnetin-glucoside	Flavonol	171.30 ± 8.57	145.76 ± 6.56
19	17.05	530, 280	639	Malvidin-coumaroyl-glucoside	Anthocyanin	n.d.	23.21 ± 1.39
20	17.18	360, 255	449, 287	Kaempferol-glucoside	Flavonol	129.78 ± 7.14	109.82 ± 4.39
21	21.35	360, 250	303	Quercetin	Flavonol	9.43 ± 0.47	23.21 ± 1.32
22	23.22	360, 260	317	Isorhamnetin	Flavonol	27.85 ± 1.67	55.71 ± 2.79
				Total phenolics		10,347.06 ± 480.89	5701.114 ± 286.77

Values are expressed as mean ± SD; n.d.—not determined.

**Table 2:** Comparison of ECG measurements recorded between experimental groups.

Groups	RR (ms)	VR (Beats/m)	PR (ms)	QRS (ms)	QT (ms)	QTc (ms)	Amp_R (mm)
**Saline**	213.00 ^a^ (203.75, 231.25)	281.50 ^a^ (259.50, 294.75)	43.50 (42.75, 45.00)	40.00 (36.75, 41.00)	80.00 (74.75, 83.50)	85.50 (79.75, 89.50)	2.50 ^a^ (2.27, 2.62)
**Saline+ISO**	151.00 (137.75, 167.25)	397.00 (358.50, 436.00)	40.00 (39.75, 43.00)	36.00 (33.75, 37.75)	87.00 (79.75, 95.50)	94.00 (86.75, 103.50)	0.85 (0.70, 1.00)
**Ramipril+ISO**	187.00 ^b^ (151.00, 191.00)	321.00 ^b^ (314.00, 399.50)	42.00 (40.50, 45.00)	35.00 (33.00, 41.00)	91.00 (89.50, 95.50)	99.00 (96.00, 103.00)	1.10 ^b^ (1.05, 1.55)
**WGP+ISO**	152.50 ^b^ (140.25, 195.75)	394.00 ^b^ (307.50, 428.25)	39.50 (38.25, 42.50)	33.50 (30.25, 35.75)	83.50 (82.25, 86.75)	90.50 (89.25, 93.50)	1.70 ^a,b^ (1.52, 1.95)
**RGP+ISO**	163.50 ^b^ (156.25, 174.25)	367.00 ^b^ (344.50, 384.00)	41.50 (39.25, 45.00)	36.00 (33.25, 39.50)	83.50 (73.75, 90.00)	89.50 (79.75, 97.00)	2.00 ^a^ (1.82, 2.80)

Values for each ECG measurement represent the median and 25–75 percentiles, where ^a^ had *p* < 0.05 versus Saline+ISO, ^b^ had *p* < 0.05 versus Saline—Kruskal–Wallis Test.

**Table 3:** Comparison of oxidative stress markers between experimental groups.

Groups	MDA (mmol/L)	NO (mmol/L)	TOS (mmol H_2_O_2_/eq/L)	TAC (mmolTrolox eq/L)	OSI	Thiols (mmol/L)
**Saline**	2.91 ^a^ (2.75, 3.02)	44.95 ^a^ (42.45, 49.22)	4.79 ^a^ (4.20, 4.98)	1.08892 ^a^ (1.08842, 1.08906)	0.50 ^a^ (0.40, 0.53)	0.0501 (0.0412, 0.0538)
**Saline+ISO**	3.56 (3.39, 4.03)	79.41 (69.10, 83.02)	7.00 (6.28, 8.44)	1.08764 (1.08743, 1.08786)	1.11 (0.97, 1.52)	0.0405 (0.0347, 0.0451)
**Ramipril+ISO**	3.52 ^b^ (3.45, 3.65)	76.98 ^b^ (72.89, 78.05)	6.87 ^b^ (5.37, 7.33)	1.08821 ^a^ (1.08768, 1.08903)	0.84 ^a,b^ (0.70, 1.07)	0.0399 (0.0362, 0.0466)
**WGP+ISO**	3.87 ^b^ (3.74, 4.13)	75.73 ^b^ (71.02, 76.47)	7.39 ^b^ (5.70, 8.57)	1.08934 ^a,c^ (1.08807, 1.08991)	1.02 ^b^ (0.87, 1.07)	0.0509 ^a,c^ (0.0465, 0.0513)
**RGP+ISO**	3.17 ^a,c,d^ (2.65, 3.36)	58.35 ^a,c,d^ (51.87, 67.41)	6.35 ^b^ (5.37, 7.20)	1.08793 ^a^ (1.08778, 1.08899)	0.93 ^b^ (0.70, 1.00)	0.0583 ^a,b,c^ (0.0533, 0.0612)

Values for each oxidative marker represent the median and 25–75 percentiles, ^a^ had *p* < 0.05 versus Saline+ISO, ^b^ had *p* < 0.05 versus Saline, ^c^ had *p* < 0.05 versus Ramipril+ISO, ^d^ had *p* < 0.05 versus WGP+ISO—Kruskal–Wallis Test.

## Data Availability

The original contributions presented in this study are included in the article. Further inquiries can be directed to the corresponding author.

## Abbreviations

The following abbreviations are used in this manuscript:

WGP	White grape pomace
RGP	Red grape pomace
GP	Grape pomace
CI	Cardiac ischemia
ISO	Isoproterenol
ECG	Electrocardiogram
TPC	Total phenolic content
DPPH	2,2-diphenyl-1-picrylhydrazyl
MDA	Malondialdehyde
NO	Nitric oxide
THIOL	Total thiols
TOS	Total oxidative stress
TAC	Total antioxidant capacity
OSI	Oxidative stress index
IL-1β	Interleukin 1β
IL-6	Interleukin 6
TNF-α	Tumor necrosis factor alpha

## Appendix A

**Table A1:** Normal distribution of rat weights.

Groups	Skewness	Shapiro–Wilk
**I—Saline**	0.165	0.210
**II—Saline+ISO**	0.276	0.694
**III—Ramipril+ISO**	0.281	0.796
**IV—WGP+ISO**	0.344	0.807
**V—RGP+ISO**	0.209	0.655

**Table A2:** Analysis of baseline weight differences among rat groups.

Groups	Baseline Mean Weight (g) ± SD	*p*
**I—Saline**	198.18 ± 27.863	0.987
**II—Saline+ISO**	204.00 ± 32.643	
**III—Ramipril+ISO**	205.50 ± 32.952	
**IV—WGP+ISO**	201.00 ± 28.265	
**V—RGP+ISO**	202.50 ± 37.212	

**Table A3:** Analysis of weight differences among rat groups on day 13.

Groups	Final Mean Weight (g) ± SD	*p*
**I—Saline**	222.27 ± 31.966	0.285
**II—Saline+ISO**	230.50 ± 29.198	
**III—Ramipril+ISO**	207.00 ± 36.148	
**IV—WGP+ISO**	202.50 ± 33.932	
**V—RGP+ISO**	203.13 ± 42.673	

**Table A4:** Serum values of the determined inflammatory markers.

Groups	TNF-α (pg/mL)	IL-6 (pg/mL)	IL-1β (pg/mL)
**Saline**	84.49 ^a^ (67.63, 137.70)	84.28 ^a^ (73.94, 121.82)	132.81 ^a^ (106.167, 166.182)
**Saline+ISO**	440.13 (286.10, 565.31)	217.66 (169.91, 375.01)	229.94 (178.990, 252.505)
**Ramipril+ISO**	59.96 ^a,d,e^ (48.76, 83.55)	93.41 ^a^ (83.13, 115.93)	107.93 ^a^ (95.297, 166.985)
**WGP+ISO**	96.83 ^a^ (83.89, 172.84)	105.24 ^a^ (98.19, 133.10)	108.36 ^a^ (72.457, 128.150)
**RGP+ISO**	103.32 ^a^ (93.58, 138.80)	113.43 ^a^ (95.25, 138.07)	125.74 ^a^ (104.925, 153.845)

Values for each inflammatory marker represent the median and 25–75 percentiles, ^a^ had *p* < 0.05 versus Saline+ISO, ^d^ had *p* < 0.05 versus WGP+ISO, ^e^ had *p* < 0.05 versus RGP+ISO—Kruskal–Wallis Test.

**Table A5:** Heart homogenate values of the determined inflammatory markers.

Groups	TNF-α (pg/mL)	IL-6 (pg/mL)	IL-1β (pg/mL)
**Saline**	28.25 ^a^ (24.50, 34.00)	20.72 ^a^ (18.85, 22.10)	9.08 ^a^ (8.44, 10.02)
**Saline+ISO**	114.37 (95.86, 143.30)	97.92 (90.15, 110.13)	48.94 (45.48, 51.08)
**Ramipril+ISO**	63.79 ^a,b^ (59.25, 75.01)	24.22 ^a,b^ (22.85, 29.96)	11.37 ^a,b^ (11.06, 14.52)
**WGP+ISO**	63.66 ^a,b^ (56.97, 105.85)	21.47 ^a^ (19.82, 29.81)	9.97 ^a^ (9.17, 11.70)
**RGP+ISO**	100.42 ^b^ (65.40, 111.51)	31.73 ^b^ (24.19, 34.02)	13.20 ^b^ (10.62, 14.98)

Values for each inflammatory marker represent the median and 25–75 percentiles. ^a^ had *p* < 0.05 versus Saline+ISO, ^b^ had *p* < 0.05 versus Saline—Kruskal–Wallis Test.

**Table A6:** Liver homogenate values of the determined inflammatory markers.

Groups	TNF-α (pg/mL)	IL-6 (pg/mL)	IL-1β (pg/mL)
**Saline**	102.42 (87.64, 116.13)	27.56 (24.35, 32.60)	13.76 (11.10, 17.98)
**Saline+ISO**	73.12 (57.86, 95.69)	22.64 (18.24, 25.92)	9.92 (8.64, 12.25)
**Ramipril+ISO**	79.62 (63.51, 105.25)	27.33 (25.46, 30.75)	12.15 (9.88, 13.67)
**WGP+ISO**	111.57 (75.75, 116.12)	27.38 (22.43, 28.94)	12.06 (10.59, 14.80)
**RGP+ISO**	80.90 (64.22, 102.30)	26.74 (25.87, 30.56)	13.92 (11.01, 16.37)

Values for each inflammatory marker represent the median and 25–75 percentiles.—Kruskal–Wallis Test.
